# Case report: Right coronary artery to left ventricular fistula complicated with coronary artery dilation presenting as persistent cardiogenic ischemic chest pain

**DOI:** 10.3389/fcvm.2023.1238034

**Published:** 2023-09-13

**Authors:** Yuehai Wang, Yuqiang Zhang, Fei Wang, Yuzeng Xue

**Affiliations:** ^1^ Cardiology Department, Liaocheng People’s Hospital of Shandong University and Liaocheng Hospital Affiliated to Shandong First Medical University, Liaocheng, China; ^2^ Laboratory Animal Center, Liaocheng People’s Hospital of Shandong University and Liaocheng Hospital Affiliated to Shandong First Medical University, Liaocheng, China; ^3^ Department of Cardiology, Shandong Corps Hospital of Chinese People’s Armed Police Forces, Jinan, China

**Keywords:** right coronary artery fistula, ischemic chest pain, coronary artery dilation, steal phenomenon, β-blocker, COVID-19, endothelin-1

## Abstract

We reported a patient with a fistula of the right coronary artery to the left ventricle, accompanied by dilation of the right coronary artery and persistent chest pain. This patient underwent surgical fistula closure surgery, but the fistula recurred. Persistent chest pain reappeared after encountering COVID-19 infection. We analyzed the mechanism of persistent myocardial ischemic chest pain caused by coronary artery fistula in this patient, the impact of surgery on the patient's disease, the possible mechanism of COVID-19 causing persistent ischemic chest pain in this patient, and the possible mechanism of metoprolol in alleviating myocardial ischemic chest pain in this patient.

## Background

Coronary artery fistula (CAF) is a rare cardiovascular disease, with an incidence rate of 0.9% (1). Most of CAFs are found accidentally in coronary angiography (2). Right coronary artery fistula (RCAF) is more common than left coronary artery fistula (2), and the receiving chamber usually includes the right ventricle (45%), the right atrium (25%) and the pulmonary artery (20%) (3), while it is rare to include the left atrium or left ventricle (LV) (4, 5). The etiology of CAF includes congenital, traumatic or iatrogenic (4, 6). Large CAFs can lead to congestive heart failure, myocardial infarction, pulmonary hypertension, etc (2, 5, 7). Patients usually complain of dyspnea, palpitation and chest pain (6, 8). Closure of fistula is usually considered as the top priority treatment option (2).

## Case present

**Figure 1:**
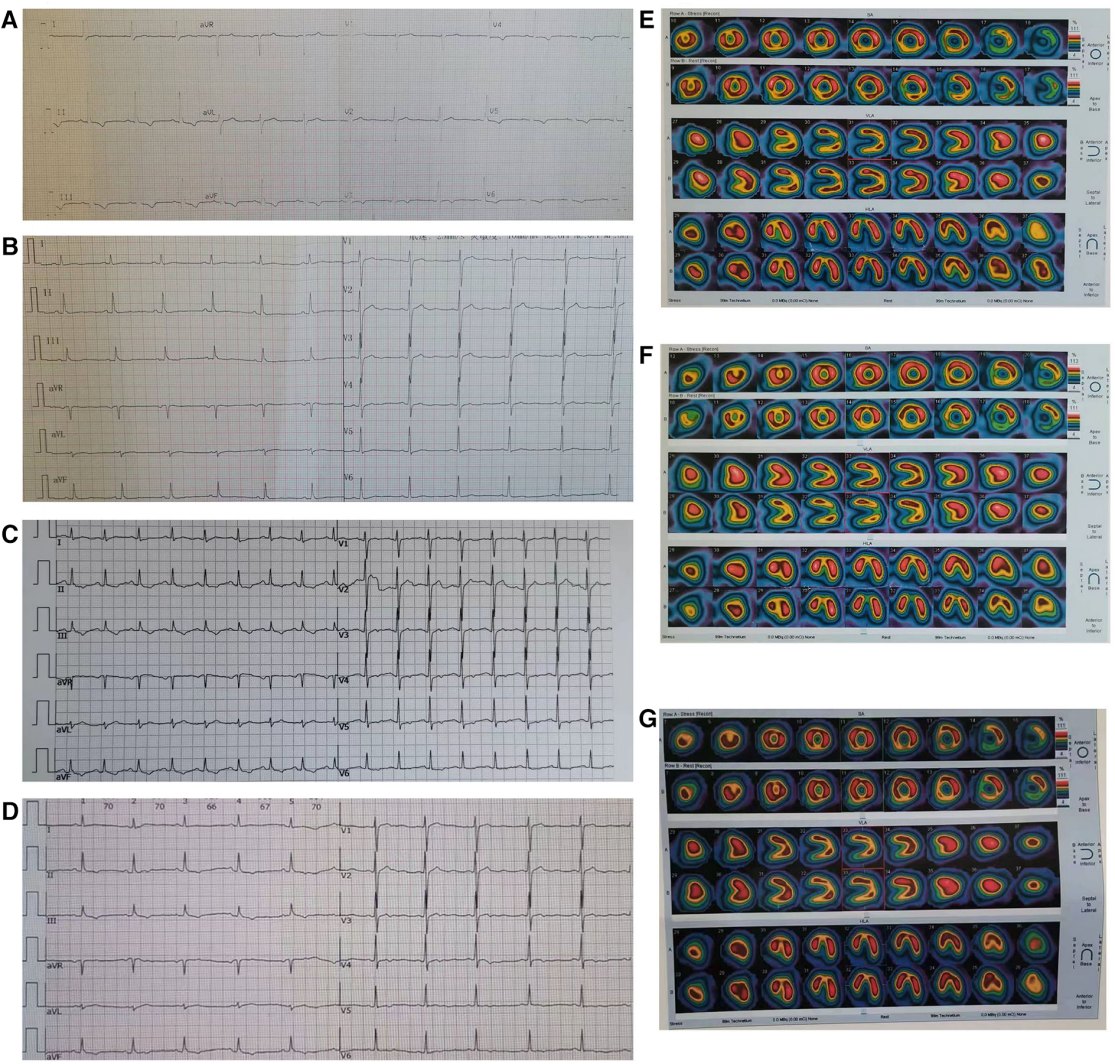
Electrocardiograms and computed tomography myocardial perfusion imaging (CTP). (**A**) T_II, III, AVF_ inversion was found in electrocardiogram before surgery; (**B**) T_II, III, AVF_ inversion disappeared in electrocardiogram after surgery; (**C**) After the patient suffered from COVID-19, the ECG showed sinus tachycardia, and T_II, III, AVF_ inversion again; (**D**) After increasing the therapeutic dose of metoprolol, the heart rate of this patient decreased from 105 beats per minute to 70 beats per minute, and the degree of inversion of T_II, III, AVF_ significantly improved; (**E**) CTP in resting and exercise states showed hypoperfusion of inferior wall myocardium before surgery; (**F**) CTP in the patient's resting and exercise states showed the disappearance of myocardial hypoperfusion of inferior wall after surgery; (**G**) After COVID-19 infection, there was no abnormal myocardial blood flow perfusion in the patient's resting and exercise states.

In the emergency department, a 33-year-old man complained of persistent chest pain with chest tightness for 24 h, and complained about the excessive work pressure in those days. There was no history of hypertension, diabetes and smoking, and no family history of early onset cardiovascular disease. The blood pressure was 106/68 mmHg, there was no lung rale and heart murmur, and the heart rate was 72 beats per minute (bpm). The electrocardiogram (ECG) showed inverted T_II, III, AVF, V3−V6_ (Figure 1A). The serum troponin I, D-dimer, N-terminal pro-B type natriuretic peptide (NT-proBNP) and cholesterol titers were normal. The patients received medication including metoprolol, aspirin and clopidogrel, with daily doses of 25 mg, 100 mg, and 75 mg, respectively. In order to determine whether the coronary artery is narrow or not, the patient underwent coronary angiography (CAG). As a result, we unexpectedly discovered a fistula from the right coronary artery (RCA) to the LV cavity, showing a rapid flow of contrast agent from RCA into LV during diastole (Supplementary Material 1, Figure 2A), and the RCA was significantly dilated, especially at the distal end of the coronary artery (Supplementary Material 1, Figures 2B,C). Computed tomography myocardial perfusion imaging (CTP) in resting and exercise states indicated myocardial ischemia in the middle and base of the LV inferior wall (Figure 1E). Transthoracic echocardiography (TTE) showed that the diameter of the orificium fistulae in the LV lumen was 4.5 mm. A bidirectional blood flow spectrum was detected at the orificium fistulae. During diastole and systole, the maximum blood flow velocity (*V*_max_) was 213 centimeters per second (cm/s) and 293 cm/s, respectively, and the maximum pressure gradient (PG_max_) was 18 millimeters of mercury (mmHg) and 34 mmHg, respectively. At the same time, in the middle and base of the LV inferior wall, hypokinesia was detected.

**Figure 2:**
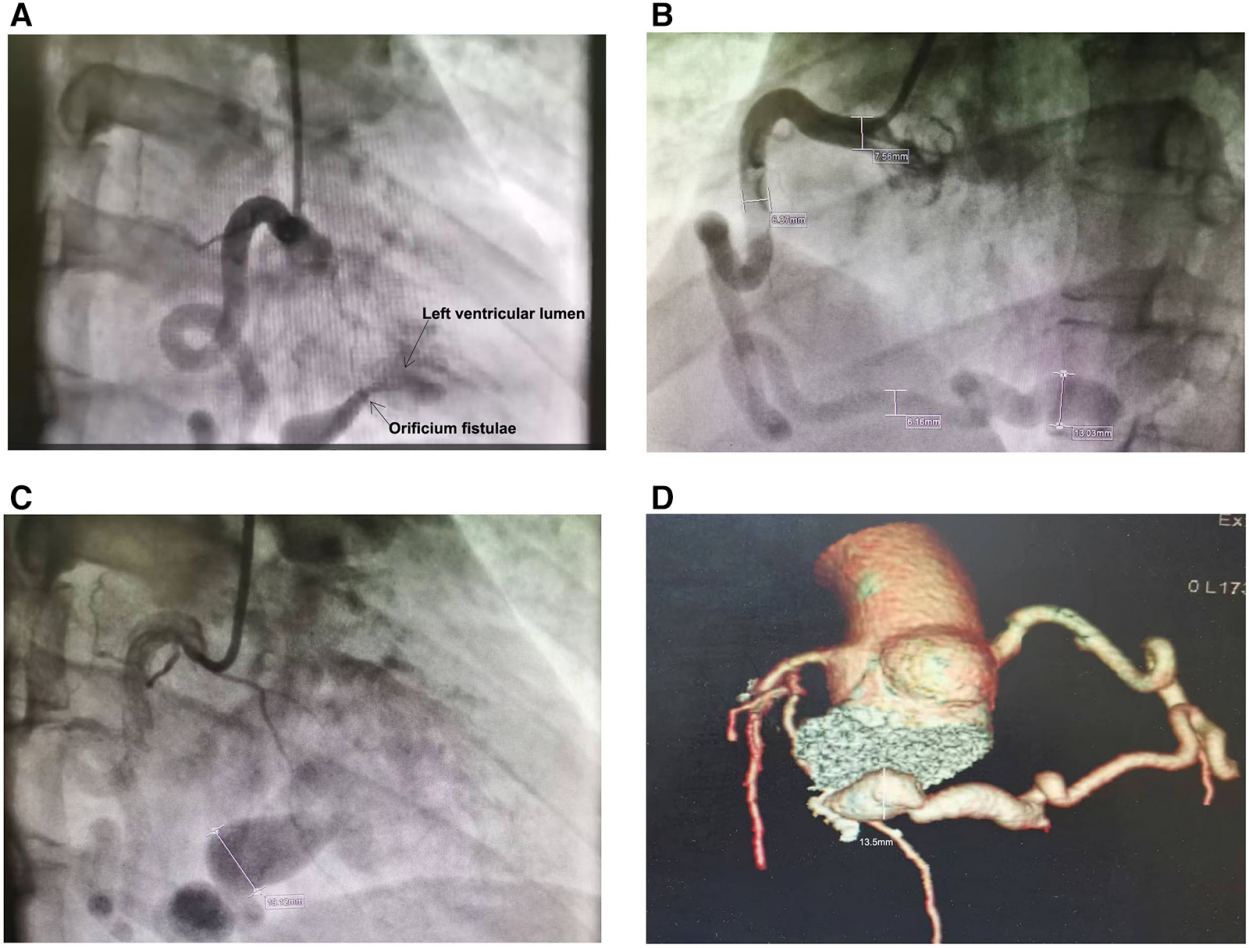
Right coronary artery to left ventricular fistula complicated with coronary artery dilation. The contrast agent flowed directly into the left ventricular lumen through the right coronary artery fistula, and the arrows point to the left ventricular lumen and orificium fistulae respectively (**A**) The lumen diameter of the right coronary artery is 7.56 mm in the proximal part (**B**), 6.37 mm in the middle part (**B**) and 19.12 mm in the distal part (**C**). In the sixth year after surgery, Coronary artery computed tomography indicated that the diameter of the distal coronary artery aneurysm in the patient's RCA was 13.5 mm (**D**).

The patient received surgical treatment. During the operation, thrill was found in the dilated RCA, and the thrill disappeared after the fistula was sutured with 3-needle 3-0 suture with felt. Before suturing the fistula, transesophageal ultrasound showed that the diameter of the fistula in the LV was 4.7 mm, the *V*_max_ was 265 cm/s, and the PG_max_ was 28 mmHg. After the fistula was sutured, the abnormal blood flow signal disappeared. However, on the third day after the operation, the TTE detected that the LV wall near the posterior mitral annulus had echogenicity from the patch, and there was a CAF on the back of the patch, indicating that the RCAF recurred. The patient had no cardiac symptoms and was discharged on the 7th day after operation. Since then, many TTEs have found diastolic blood flow signals at the outlet of recurrent fistula, but no systolic blood flow signals. The mean values of diameter, *V*_max_ and PG_max_ at the orificium fistula in diastole were 3.5 mm, 210 cm/s and 18 mmHg, respectively. Low amplitude T_II, III, AVF_ were found on the ECG (Figure 1B), and CTP did not reveal myocardial ischemia (Figure 1F). In the next five years, the patient occasionally had slight chest tightness but no chest pain. After taking metoprolol 25 mg per day, the symptoms were significantly relieved, and the frequency of attack was significantly reduced. The patient insisted on taking metoprolol for most of the time, and the related data of CAF in the patient had no significant change detected by TTE.

However, on December 24, 2023, the patient suffered from Coronavirus Disease 2019 (COVID-19), developed fever and cough, and the COVID-19 test was negative 10 days later. Since then, the patient has intermittent mild palpitation, chest tightness and chest pain. After taking metoprolol, the palpitation can be reduced. After 1 month, the patient suffered from persistent chest pain and chest tightness again and went to the hospital again. The chest pain could affect the precordial area, back and left shoulder. Heart rate was 105 bpm. The troponin I (TNI) and NT-proBNP titers did not increase, but the serum endothelin 1 (ET-1) titer increased to 10.95 ng/ml. The ECG showed sinus tachycardia and inverted T_II, III, AVF_ (Figure 1C). TTE showed that in the diastole, the diameter of the orificium fistula was 2.5 mm, and the the *V*_max_ was 145 cm/s. Myocardial ischemia was not detected by CTP (Figure 1G). Coronary artery computed tomography scan revealed a diameter of 13.5 mm for a distal coronary artery aneurysm (CAA) in the RCA (Figure 2D). The efficacy of metoprolol at a daily dose of 25 mg was poor, and metoprolol was increased to 50 mg per day, and the heart rate decreased from 105 bpm to 70 bpm, accompanied by a significant improvement in the degree of T_II, III, AVF_ inversion (Figures 1C,D; Table 1). After 5 days, chest tightness and pain relieved.

**Table 1:** Timeline of the changes in patient symptoms, auxiliary examination results, and treatment.

	October 25, 2017	November 9, 2017	November 12, 2017	December 12, 2018	December 24, 2023	January 3, 2024	January 8, 2024
Symptom	Persistent chest pain with chest tightness	No	No	No	Intermittent chest pain, palpitation, and chest tightness	Persistent chest pain with chest tightness	No
Changes in T waves on ECG	Inverted T_II, III, AVF, V3−V6_		Low amplitude T_II, III, AVF_			Inverted T_II, III, AVF_	Improvement of T_II, III, AVF_ inversion
Tachycardia	No	No	No	No		Yes	No
Indicated myocardial ischemia by CTP	Yes		No			No	
Diameter of the orificium fistulae in the LV lumen	4.5 mm	0	3.5 mm	3.6 mm		2.5 mm	
Bidirectional blood at the orificium fistulae	Yes	No	No	No		No	
*V*_max_-1	213 cm/s	0	210 cm/s	198 cm/s		145 cm/s	
*V*_max_-2	293 cm/s	0	0	0		0	
Diameter of d-RCA	19.12 mm					13.5 mm	
PG_max_ in diastole	18 mmHg	0	18 mmHg	16 mmHg			
PG_max_ in systole	34 mmHg	0	0	0			
Metoprolol	25 mg/d	25 mg/d	25 mg/d	25 mg/d	25 mg/d	50 mg/d	50 mg/d
Surgical treatment		Yes					
Recurrence of CAF			Yes				
ET-1 titer						Increased	

ECG, electrocardiogram; CTP, computed tomography myocardial perfusion imaging; LV, left ventricle; *V*_max_-1, the maximum blood flow velocity at the outlet of the coronary artery fistula detected by transthoracic echocardiography during the diastolic phase of the heart; *V*_max_-2, the maximum blood flow velocity at the outlet of the coronary artery fistula detected by transthoracic echocardiography during cardiac systole. cm/s, cm per second; d-RCA, distal part of right coronary artery; PG_max_, maximum pressure gradient; mg/d, mg per day; CAF, coronary artery fistula; ET-1, endothelin 1.

## Discussion

### Etiology

For this patient, the latter two can be excluded from the three major causes of congenital, traumatic, or iatrogenic diseases.

### Coronary ectasia

Coronary artery atherosclerosis, Kawasaki disease, coronary artery dysplasia and trauma can lead to coronary artery aneurysm (9). However, the above reasons cannot explain the coronary artery dilation and CAA formation in this CAF patient. In diastole, LV pressure drops significantly to 0–10 mmHg, which leads to the rapid flow of blood from the main branch of RCA on the surface of the heart into the LV through the orificium fistula. The long-term impact of this rapid blood flow on the coronary artery wall may be the pathological reason for the dilation of the RCA. The patient's RCA dilated to 7.56 mm, 6.37 mm, and 19.12 mm in the proximal, middle, and distal parts (Figures 2B,C), respectively. A huge aneurysm had already formed in the distal end of the RCA. Preoperative TTE showed that there was bidirectional blood flow at the orificium fistulae. On both sides of the orificium fistula, the systolic PG_max_ and *V*_max_ were greater than those in diastole. This results in a stronger impact of systolic blood flow from LV on the distal end of RCA compared to diastolic blood flow from the coronary artery. Therefore, the formation of a distal CAA in this patient's RCA is more closely related to the systolic blood flow impact, which disappeared with surgical treatment, possibly resulting in a reduction in the diameter of the CAA to 13.5 mm five years after surgery (Figure 2D; Table 1).

### Mechanism and symptoms of myocardial ischemia

In the diastole of the heart, due to the obvious pressure difference between the RCA orifice and the LV chamber, the blood in the RCA of this patient rapidly and directly flows into the LV chamber through the fistula. As the RCA gradually expands, it becomes easier for the blood in the lumen to flow into the LV chamber, so that the decrease of myocardial blood perfusion in the RCA distribution area gradually intensifies. This can be referred to as “stealing blood”. Under certain inducing factors of increased myocardial oxygen consumption, myocardial ischemia and hypoxia suddenly worsen, leading to myocardial ischemia symptoms.

The common symptoms of CAF include dyspnea, palpitations, and chest pain (6, 8). This patient complained of persistent chest pain during his first visit to the doctor. The ECG, TTE, and CTP all indicate myocardial ischemia, therefore we conclude that the patient has persistent ischemic chest pain. The cause of myocardial ischemia is “stealing blood” caused by CAF, and the increased demand for blood in the myocardium due to work pressure may be a trigger for symptoms of myocardial ischemia. The increased pressure can lead to sympathetic nerve activity. It remains to be further clarified whether this patient has an increase in sympathetic amine substances that induce endothelial dysfunction or spasms in small coronary arteries.

However, persistent ischemic chest pain did not result in an increase of TNI, a marker of myocardial injury. This may be related to the insufficient degree of ischemia and the absence of stenotic lesions in the branches of RCA.

The patient underwent surgical treatment and the fistula was sealed with a patch. Although the fistula recurred again after surgery, there was no persistent chest pain within the next 5 years, the inverted T wave in the ECG disappeared (Figure 1B), and CTP did not detect myocardial hypoperfusion (Figure 1F). This indicates that the patient's myocardial ischemia has been significantly improved. Although the patient's CAF reappeared after being occluded, the orificium fistula became smaller, and the patch to some extent blocked blood flow and changed the direction of blood flow and reduced blood flow velocity, resulting in a decrease in total blood volume entering the LV, ultimately reducing “stealing blood”. In addition, in the sixth year after operation, the orificium fistula was further reduced to 2.5 mm, and the *V*_max_ was further reduced to 145 cm/s in diastole (Table 1). This indicates that the CAF is gradually decreasing and “stealing blood” is gradually decreasing.

### Chest pain after COVID-19 infection in this patient

After COVID-19 infection, this patient experienced persistent chest pain accompanied by referred pain, tachycardia, and T-wave inversion again, but did not show insufficient myocardial blood flow perfusion (Figure 1G) as indicated by CTP. Additionally, the depth of the inverted T-wave was not as significant as before surgery under the premise of similar heart rate (Figures 1A,D), and TTE indicated that the patient's CAF blood stealing phenomenon did not worsen. All of these manifestations suggest that the patient experienced mild myocardial ischemia. Undoubtedly, the “stealing blood” caused by recurrent RCAF makes the myocardium more prone to suffer from symptomatic myocardial ischemia under the influence of other triggers, which may include increased sympathetic nervous activity and elevated serum ET-1 titer after COVID-19 infection. Previous studies have suggested that some COVID-19 patients experience chest pain (10), chest tightness, fatigue, anxiety and high heart rate in the following months (11, 12). Some cases of chest pain resemble angina pectoris, while others present as persistent chest pain (13). This ischemic symptom may be related to coronary microvascular perfusion defects (13), which can last for one to six month (11, 14) and may be related to angiotensin converting enzyme 2 mediated endothelial damage, microvascular inflammation, thrombosis, and endothelial dysfunction (15, 16). There are also studies showing an increase in serum ET-1 titer in COVID-19 patients (17, 18), which is related to coronary microvascular endothelial dysfunction (19, 20).

The patient had chest symptoms after virus infection, so we need to differentiate it from viral myocarditis. However, this patient did not exhibit elevated biomarkers of myocardial injury or heart failure, as well as symptoms of heart failure or severe arrhythmia. Therefore, we excluded the diagnosis of myocarditis. In addition, pulmonary embolism should be differentiated. However, the patient had no evidence of venous thrombosis, no elevated titer of D-dimer, no S_I_Q_III_T_III_ and elevated pulmonary artery pressure. Therefore, we also excluded this disease.

### Treatment

#### Interventional and surgical treatment

Patients with significant left to right shunt, heart failure accompanied by LV volume overload or LV dysfunction, and myocardial ischemia can only receive catheter intervention embolization or surgical repair for CAF (2).

The outlet of CAF is located in the LV, and the flow rate of blood through the fistula is fast. If this patient receives interventional embolization treatment, the risk of developing cerebral embolism and other systemic embolism is high. This patient experienced a recurrence of CAF after undergoing fistula closure surgery, but due to the reduction of “stealing blood”. the patient's symptoms were significantly improved. Meanwhile, the degree of coronary artery dilation near the fistula outlet was improved (Figures 2C,D) due to the disappearance of blood flow at the fistula outlet during systole.

### Metoprolol

At the patient's first visit, oral metoprolol can alleviate chest pain. Later, when the patient experiences COVID-19 infection, chest pain can also be alleviated by increasing the dosage of metoprolol. This suggests that metoprolol can alleviate ischemic chest pain in this CAF patient. We know that metoprolol can be used to treat coronary artery disease by reducing the blood demand of the myocardium by reducing heart rate and myocardial contractility, and this mechanism can also alleviate the degree of myocardial ischemia caused by “stealing blood” in CAF patients.

In addition, we know that the diastole is divided into isovolumic relaxation, rapid filling and slow filling phases. Metoprolol can prolong diastole by reducing the heart rate, thereby prolonging the slow filling phase. During the slow filling phase, the increase in blood volume in the LV leads to an increase in pressure, resulting in a decrease in the velocity of blood flowing into the LV through the RCAF of this patient. Therefore, metoprolol can reduce the “stealing blood” from CAF by reducing heart rate and prolonging the slow filling phase, thereby improving the blood perfusion of microvessels in the RCA branch.

## Data Availability

The original contributions presented in the study are included in the article/**Supplementary Material**, further inquiries can be directed to the corresponding author.

## Supplementary material

Supplement Material 1 Right coronary artery to left ventricular fistula complicated with coronary artery dilation found in coronary angiography.
